# Long-Term Outcome of Transcatheter Device Closure of Perimembranous Ventricular Septal Defects

**DOI:** 10.3389/fped.2018.00128

**Published:** 2018-05-03

**Authors:** Krishna D. Mandal, Danyan Su, Yusheng Pang

**Affiliations:** Department of Pediatrics, The First Affiliated Hospital of Guangxi Medical University, Nanning, China

**Keywords:** cardiac catheterization, congenital heart defect, device closure, outcomes, ventricular septal defect

## Abstract

**Background:** Ventricular septal defect is a common congenital heart defect. Transcatheter closure of perimembranous ventricular septal defect (pmVSD) is an effective method alternative to surgical closure. The aim of the study is to evaluate the procedural result, early and long-term follow-up outcome of transcatheter closure of pmVSD.

**Methods:** From January 2005 to December 2016, we retrospectively identified the patients who underwent transcatheter device closure of pmVSD. All patients underwent transthoracic echocardiography (TTE) and electrocardiogram (ECG) before and after the procedure. Follow-up evaluation was done at 1, 3, 6, 12 months and yearly thereafter with TTE and ECG.

**Results:** Total 186 patients (95 male, 91 female) had catheter-based intervention of pmVSD. The mean age at the time of procedure was 5.4 ± 2.8 years (range 2~14 years) and the mean weight was 18.1 ± 6.7 kg (range 10.5~43 kg). The mean size of the implanted device was 8.4 ± 2.1 mm (range 5~16 mm). The device was successfully implanted in 180 patients (96.8%). Immediate post-procedural echocardiography showed complete occlusion in all but one patient had trivial residual shunt. Total early adverse events occurred in 16 patients (8.9%). Only in two patients it was significant, complete AVB occurred in a 9 years old boy, managed with temporary pacemaker and one patient had complete left bundle branch block, recovered fully after steroid therapy. During a median follow-up period of 18.4 months (range 6~120 months), no serious adverse events and complete AVB were encountered.

**Conclusion:** In our experience, The incidence of serious adverse event is low and no late onset of complete AVB with excellent success rate and follow-up results, confirming the transcatheter closure of pmVSD is a valuable alternative to surgical closure in selected patients.

## Introduction

Despite medical and surgical therapy, congenital heart disease (CHD) is the one of the leading cause of morbidity and mortality from a birth defect. It affects ~0.8% of live birth (1). Due to advance in cardiac imaging modalities and techniques, more type of CHD can be treated in cardiac catheter laboratory. However, transcatheter device closure of perimembranous ventricular septal defect (pmVSD) remains challenging procedure due to proximity of the defect to the aortic and tricuspid valves as well to the conduction system. Therefore, there is Possibility of device influencing the aortic valve (2), and interfering the conduction system (3–6). Complete atrioventricular block (AVB) is the most serious complication related to the procedure and device, which can occur either during or any time after the procedure (5–8). Published literature demonstrated that variety of device has been used to close the pmVSD. The immediate and mid term result of percutaneous closure of pmVSD has been documented previously (5, 7, 9, 10). However, long-term results are missing. In the present study, we sought to evaluate the procedural result, early and long-term follow-up outcomes of transcatheter device closure of PmVSD.

## Materials and methods

From January 2005 to December 2016, we retrospectively identified the patients diagnosed with pmVSD who underwent percutaneous device closure of pmVSD at First Affiliated Hospital of Guangxi Medical University. Written informed consent was obtained from the patient's parents, and the medical ethics committee of the hospital approved the study. All patients were assessed by transthoracic echocardiography (TTE) (iE33, Philips medical system, Andover, Massachusetts), including M-mode, two dimensional, and color Doppler examination. Size and types of VSD were measured by a standard four-chamber view, five-chamber view, and parasternal long and short axis view. Only patients with minimal weight of 10 kg and age ≥2 years old were qualified for the procedure. Following inclusion criteria were used in this study: TTE evidence of left ventricular and/ or left atrial enlargement, pmVSD located at the 9–11 o'clock position of an analog clock in the short axis parasternal view with significant left to right shunt through the pmVSD, pulmonary to systemic blood flow ratio (QP/QS) >1.5, cardiomegaly on chest x-ray, frequent respiratory infection (six events per year), failure to thrive according to literature (6), and New York Heart Association function class II or greater.

Patients with pmVSD prolapse aortic cups, pmVSD with infundibular defect, mal-alignment, rims ≤1 mm under the aortic valve, active endocarditis, contraindication to antiplatelet or anticoagulation therapy, severe pulmonary hypertension or pulmonary vascular resistance >8 wood units, and VSD associated with other congenital cardiac anomalies which need surgical treatment were excluded from the study.

Preoperative routine examination including standard 12 leads electrocardiogram (ECG), chest X-ray, TTE, and blood test was done in all patients. Patient's general characteristics are reported in Table 1.

**Table 1:** Patients general characteristic, age distribution and their indication for transcatheter procedure.

**Patients (n)**	**186**
Male	95 (51.1%)
Female	91 (48.9%)
Age (years)	5.4 ± 2.8 (2–14)
**AGE GROUP (n)**	
<5 years	96 (51.6%)
5–10 years	75 (40.3%)
> 10 years	15 (8.1%)
Weight (kg)	18.1 ± 6.7(10.5–43)
**INDICATION**	
Symptoms (frequent respiratory infection, edema, NYHA function class II or grater)	89 (47.8%)
Hemodynamic changes (cardiomegaly in chest x-ray, left atrial or ventricle enlarge by TTE)	97 (52.1%)

*NYHA; New York Hear Association; mean ± standard deviation (range) (percentage)*.

### Device and delivery system

**Figure 1:**
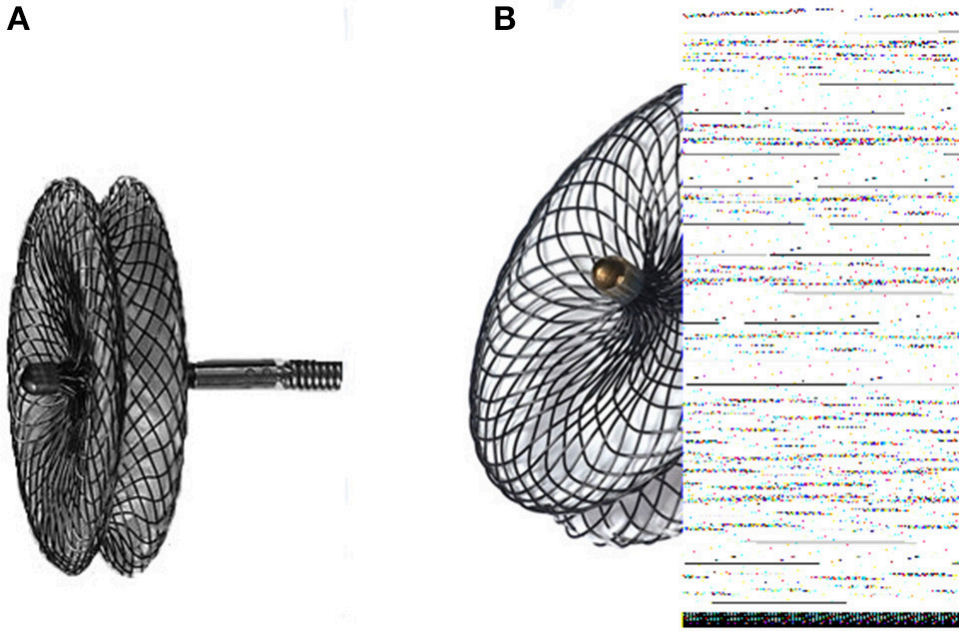
**(A)** Shanghai perimembranous ventricular septal defect occluder; **(B)** Lifetech Shenzhen perimembranous ventricular septal defect occluder.

All the device and delivery systems were used in this study made by Lifetech Scientific Shenzhen Co. Ltd (Shenzhen, China) and occasionally we used Shanghai pmVSD occluder made by Shanghai shape memory alloy Co. Ltd (Shanghai, China). Both the occluders are symmetrical double disk self-expandable made of 0.005-inch nitinol wire mesh and fabric inside each. The symmetric occluder was used for those defects with rim ≥2mm under the aortic valves. The diameter of both discs is 4 mm larger than that of the waist diameter and thickness of the waist of the occluder is 3 mm. The Shanghai pmVSD occluder was approved by the state Food and Drugs Administration, P.R. China, in 2003 and received trademark in 2008 (7, 11). The Lifetech Scientific Shenzhen pmVSD occluder and delivery system has been described previously (12). Shenzhen pmVSD concluder's both disc are connected by short waist, that corresponds to defect size and the device is sewn with expanded polytetrafluoroethylene to increase its closing ability (both the occluders shown in Figure 1).

### Procedure

Transcatheter closure of pmVSD was performed under general anesthesia, conscious sedation and under local anesthesia with 1% lidocaine (usually >10 years old) for the children who can coordinate. Access was through the femoral vein and artery. Heparin (100 IU/kg) and intravenous antibiotic were administrated.

The procedure was performed under fluoroscopic control. Standard right and left cardiac catheterization were performed for hemodynamic assessment in all cases. Left ventricular angiography was performed at 60° to 20° left anterior oblique projection/cranial to profile the pmVSD and angiography of ascending aorta to profile the aortic valves. Left ventriculography combine with intraoperative TTE were used to obtain the location, size of pmVSD, and its relationship with adjacent aortic valves. The diameter of pmVSD was measured at largest diastolic phage on the left ventricular side and was calculated by integrating data from the TTE and angiography measurement. The device was selected 1mm or 2 mm larger than the measured VSD diameter.

The defect was crossed from the left ventricle using partly cutting pigtail catheter and an exchange floppy guide wire was advanced into the pulmonary artery or the superior or inferior vena cava. The wire was then snared to establish an arteriovenous circuit through femoral vein approach on the same side. An appropriate size of dilator and long sheath was advanced to the left ventricle through the arteriovenous circuit and positioned beneath the aortic valve. Through the long sheath, selected occluder was deployed under fluoroscopic control. The procedure was described in detailed previously (11). After full deployment of the occluder, TTE combined with angiography were performed again to verify position and shape of the device, complete occlusion, residual shunt, and new onset of aortic valve regurgitation. Then the device was released after confirming the correct position of it and an entire delivery system was withdrawn. Patients were transferred to general wards; continuous ECG monitoring was used for 24 h after the procedure. Aspirin (3-5 mg/kg) was administered for 6 months in all patients. Clinical examination, chest x-ray, TTE, and ECG were done before the hospital discharge.

### Follow-up protocol

All the patients were follow-up at 1, 3, 6, 12 months after the procedure and yearly thereafter. Adverse events were monitored in each visit on the basis of basic clinical evaluation, chest x-ray, TTE, ECG. The patient's records were reviewed in detailed and telephone survey was attempted with the patient who did not have documented follow-up record.

### Statistics

Data are expressed as frequency or percentage for normal variables, and mean ± standard deviation for continuous variables, median, and range as appropriate. Univariate analysis was performed using chi-square test, Fisher exact test, unpaired student *t*-test and multivariable analysis to study risk factor for the occurrence of early adverse event and arrhythmia was performed using logistic regression analysis. All statistical analysis were performed by using SPSS version 16.0 for windows, *P*-value < 0.05 was considered significant.

## Results

Following the inclusion criteria total 186 patients (95 male, 91 female) had catheter-based intervention of pmVSD. The mean age at the time of procedure was 5.4 ± 2.8 years (range 2~14 years) and the mean weight was 18.1 ± 6.7 kg (range 10.5~43 kg). During the procedure, the mean QP/QS recorded as 1.82 ± 0.54 (range 1.4~4.41), the mean defect size measured by angiography was 6.9 ± 2.2 mm (range 3~14 mm), and the mean size of the implanted device was 8.4 ± 2.1 mm (range 5~16 mm). The device was successfully implanted in 180 patients (96.8%) whereas failed to implant in 6 patients (3.2%). The reason of failure was; severe residual shunt on angiography after placement of the occluder, so the device was removed before unscrewing in one patient, in a 6-years-old boy the procedure time was prolonged with excess bleeding at puncture site, and in remaining four patients due to instability, we were unable to position the device even after several attempted. so the devices were not released and procedures were aborted in all these patents. It is possible that there were an error in their patient selection and/ or device and delivery techniques. All these patients underwent uneventful surgical closure of pmVSD. Furthermore, two patients underwent combined procedure; patent ductus arteriousus closure and pulmonary balloon valvuloplasty simultaneously in each. The procedural data summarized in Table 2.

**Table 2:** Tabulation of procedural characteristics, device and defect size.

**Procedure time (min)**	**86.1 ± 29.7 (25–200)**
Fluoroscopic time (min)	20.9 ± 14.5 (2.3–83)
QP/QS	1.82 ± 0.54 (1.4–4.41)
Mean PAP (mmHg)	17.1± 5.1
Defect size by angiography (mm)	6.9 ± 2.2 (3–14)
Device size (mm)	8.4 ± 2.1 (5–16)
**TYPES OF DEVICE USED (n)**	
Shenzhen pmVSD-O	166
Shanghai pmVSD-O	14
**COMBINED PROCEDURE**	
Pulmonary valvuloplasty	1
PDA closure	1

*Min, minutes; QP/QS, pulmonary to systemic flow ratio; PAP, pulmonary artery pressure; PDA, patent ductus arteriosus, mean ± standard deviation (range); pmVSD-O, perimembranous ventricular septal defect occluder*.

### Early post procedure adverse events

In 180 (96.8%) successful procedure total 19 early adverse events occurred in 16 patients (8.9%). the most common adverse events related with pmVSD closure was rhythm disturbance (9 of 16). Only in two patients, it was significant remaining were transient; Complete AVB occurred 4 days after placement of 12mm pmVSD occluder in a 9 years old boy, the patient was managed successfully with temporary pacemaker and corticosteroid therapy, AVB recovered after 2 weeks of treatment and complete left bundle branch block (LBBB) developed in 4-years-old boy after the procedure and the patient was recovered fully after 13 days of steroid therapy.

One patient had transient complete right bundle branch block (RBBB) with T wave abnormality after the procedure and reoccurred during the follow-up.

Five patients (2.8%) experienced transient incomplete RBBB. One of five patients suffered from brady-arrhythmia with a heart rate of 57 beats per minutes. All of these patients recovered before discharge. However, one patient had recurrent incomplete RBBB during the follow-up.

Frequent atrial premature contraction with junctional escape rhythm occurred in one patient (0.6%), which resolved spontaneously before discharge.

One patient (0.6%) had new onset of mild tricuspid regurgitation (TR), who was treated with 7mm of pmVSD occluder, the regurgitation was disappeared completely in first visit.

Three patients (1.7%) needed blood transfusion due to bleeding at puncture site in one patient; hemolysis in one and hemoglobin dropped in one patient, the cause remains unknown. The transfusion volumes were less than one unit in all these patients.

Hemolysis developed in two patients (1.1%). A 6-years-old female had brown color of urine after the procedure and needed blood transfusion that was mentioned above and another 3-years-old boy who had G6PD deficiency, resolved spontaneously after stopping the aspirin.

Bleeding and groin hematoma occurred in three patients (1.7%). one patient needed blood transfusion as described above. Immediate post-procedural TTE showed complete occlusion of the defect in all patients but trivial residual shunt was seen in one patient and resolve in first visit. Summarize in Table 3.

**Table 3:** Early adverse events and their distribution.

**Events**	**No**.
Complete AVB	1
Complete LBBB	1
Complete RBBB	1
Incomplete RBBB	5
Atrial premature contraction	1
Mild TR	1
Hemolysis	2
Blood transfusion	3
Bleeding and groin hematoma	3
Residual shunt	1
Total	19

*Some patients experienced more than one early adverse event. AVB, atrioventricular block; RBBB, right bundle branch block; LBBB, left bundle branch block; TR, tricuspid regurgitation*.

In the univariate analysis, we used sex, age, weight, device diameter, defect diameter, procedure time and fluoroscopic time. However, no variable was significantly associated with the occurrence of early adverse events.

### Follow-up result

Total follow-up data were available in 175 patients (97.2%) whereas five patients (2.8%) lost the follow-up. The mean duration of follow-up was 18.4 month (range 6~120 month). No mortality and major adverse events were noted. Patients with frequent respiratory infection had no recurrence; patients with failure to thrive had recovered of growth corresponding to their age and sex; Cardiomegaly resolved in all patients and the New York Heart Association functional class returns to I in all cases.

No complete AVB occurred during the entire follow-up, however, total 17 patients (9.7%) had transient adverse events. Complete RBBB occurred in four patients (2.3%) without any clinical symptoms. It occurred early after 1 month in one patient, after 1 year in another one patient and after 3 years in remaining two patients. Nine patients (5.1%) experienced incomplete RBBB in between 6 and 12 months of follow-up, no treatment was needed. First degree AVB occurred in one patient (0.6%) after 2 years without any symptoms. All of those transient arrhythmias either resolved spontaneously or were managed with pharmacotherapy. Mild TR occurred in two patients (1.1%) after 3 months in 10 years old boy treated with 10 mm of pmVSD occluder and after 6 months in 3 years old boy treated with 7 mm of pmVSD occluder. One patient (0.6%) had aortic regurgitation (AR) after 4 years, treated with 10mm of pmVSD occluder; all of the above regurgitate volumes measured by Doppler were less than 5 ml; no evidence of enlarged heart and none of them required intervention.

## Discussion

Interventional therapy of pmVSD has been wildly used, previously cited report shown variety of devices have been used to treat the pmVSD with encouraging result. Now, the use of pmVSD device in patients, who had 1–2 mm distance between the defect and aortic valve. However, the Amplatzer MVSD-O was used in patients who had a distance of at least 5 mm between the superior rim of the defect and the aortic valve (6). Therefore, treatment option of pmVSD has been increased. Published literature reported that the rate of successful device closure of pmVSD was between 91.9 and 99% (3, 5–7, 9–13). Consistent with this, in our report the rate of successful device implantation was 96.8%. We found an aneurysm tissue in 7.5% of our patients. An aneurysm of the membranous septum we tried to close the true anatomical hole with using appropriate device size. In a case of small aneurysm, the device could cover the defect as well as the aneurysm; in a case of large aneurysm, the device was implanted entirely within the aneurysm.

Total early adverse events occurred in 8.9% whereas 9.7% during the follow-up. However, no severe adverse events complete AVB and mortality occurred during the entire follow-up in current study. This is similar to a previous report (5, 14), total early adverse events was 10.3% whereas significant adverse event was 1.3% with 32 months of follow-up and no serious adverse events during 10 years of follow-up respectively. As per others reports the total early adverse events ranges as 11.5–14.49% and serious adverse events ranges as 1.12–8.7% (3, 6, 7, 12), which is relatively higher than the current study. Recent published data of VSD closed with the Nit-Occlud® Lê VSD-Coil in 110 patients showed total adverse events occurred in 20.5% and serious events in 1.9% of patients (10). It is possible that variety of devices have been used to minimize the risk of complication.

Cardiac arrhythmia has been commonly reported in transcatheter closure of pmVSD due to the proximity of the conduction system closure to the perimembranous defect. One of serious complication is complete AVB and has been reported in 0.23–6.4% of the cases in various studies (3, 5–7, 12, 14). Complete AVB usually occurred soon after the transcatheter procedure due to squeezing effect of the device, an oversized device, and direct compression or late due to inflammation and fibrous tissue formation (6, 15). In present study complete AVB occurred 4 days after placement of 12 mm pmVSD occluder in a 9 years old boy. Initially, the patient was treated with steroid, and on 6th day, temporary pacemaker was implanted. The patient was successfully managed with both temporary pacemaker and corticosteroid therapy, after 2 weeks ECG showed normal rhythm. Possible explanation are the early AVB may be due to mobile device disc rubbing the conduction tissue in each cardiac cycle or could be the large device size (12 mm) may compress perinodal tissue which may provoke inflammation and edema, this explains why patent had early AVB and managed successfully with a temporary pacemaker and steroid therapy.

Steroid has been used in various studies to treat the complete AVB with varying success (5, 16). Steroid therapy can reduced the inflammation and edema in the perinodal tissue. There was a beneficial effect of steroid and temporary pacemaker in our patient, which suggest inflammatory cause rather than mechanical, that is similar to previous report (12, 14). As per literature, Age is significantly associated with the occurrence of complete AVB in <6 years old (5, 6), while other report showed AVB mainly noted in <3 years of age group (17). In our case, the patient was more than 5 years old. Complete AVB occurred even in surgical closure of pmVSD in 1.1% of patients, however, a recent meta-analysis of device closure vs. surgical closure of pmVSD showed no significant differences in complete AVB complication; (18, 19). Therefore surgical and device closure of pmVSD both may interfere the conduction system.

Bundle branch block including right and left branch block are quite common which may cause adverse hemodynamic outcome. Complete LBBB is relatively rare and may resolve with or without steroid therapy, in some case, it can cause chamber enlargement and heart failure (6, 8, 12, 20). In our study, CLBBB developed in 4-years-old boy early after implantation of 6mm occluder. The patient recovered fully after 13 days of steroid therapy. A mechanism is same as above. Other transient arrhythmias occurred early as well as during the follow-up, which resolved spontaneously without treatment.

Hemolysis is another serious complication usually emerged immediately after the procedure. It may transient in some case, require medication, or need blood transfusion (6, 7, 10). However, in case of massive hemolysis, that required surgical retrieval of the device (3). In our experience hemolysis occurred in two patients, blood transfusion required in one patient treated with 10 mm of pmVSD occluder and in another one patient it was transient resolved spontaneously.

New onset of valve regurgitation both aortic and tricuspid regurgitation are well known after the procedure. Valves regurgitation caused by impingement of occluder on the aortic leaflets leads to instant AR and by interfering with the chordae tendineae leads to TR (2, 3, 21). TR has seen in 5.4% of case and did not required intervention (21). However, surgical intervention required in case of severe TR (12) and progressive AR (3, 7). Thus, TTE is crucial for pre, intra, and post procedure monitoring. In present study, TR occurred in three patients, early after the procedure in one patient, in 3 and 6 months respectively in remaining two. AR was noted after 4 years of procedure in one patient, who treated with 10mm of occluder. All these patients were asymptomatic; TTE revealed no evidence of chamber enlargement and no intervention was needed during follow-up. Mechanism is not well understood. Possibly, TR was caused by interference with chordae tendineae by catheter or guide wire during the procedure or right disc of device may cause injury to tricuspid valve and AR during follow-up may cause by impingement of device on aortic leaflets.

Author experiences the risk of pediatric catheterization continue to decline, majority of adverse event in present study were self-limited only few patients required medical attendance. Careful patient selection, skillful technique, and good medical care could reduce the risk of complication.

## Study limitation

Current study has some limitation such as this is single-center retrospective study, selection bias could play role, sample size and the follow-up period could be judged to be moderate because some patient had only 6 months of follow-up, and complication related to anesthesia were failed to document.

## Conclusion

In our experience, the incidence of serious adverse event is very low and no late onset of complete AVB with excellent success and closure rate, confirming the transcatheter device closure of pmVSD is a valuable in selected patients with favorable early and long-term outcome. Conduction disorders remains a common problem; further large-scale study is needed in future to identify the potential risk factor for the occurrence of complication.

## Author contributions

MD: first author, assisting surgeon, approval of final manuscript. DS: approval of final manuscript. YP: operating surgeon participated in study design, approval of final manuscript. All authors read and approved the final manuscript.

### Conflict of interest statement

The authors declare that the research was conducted in the absence of any commercial or financial relationships that could be construed as a potential conflict of interest.

## References

[1] Bom TVD Zomer AC Zwinderman AH Meijboom FJ Bouma BJ Mulder BJM. The changing epidemiology of congenital heart disease. Nat. Rev. Cardiol. (2011) 8:50–60. 10.1038/nrcardio.2010.166 21045784

[2] Kenny D Tometzki A Martin R. Significant aortic regurgitation associated with transcatheter closure of perimembranous ventricular septal defects with a deficient aortic rim. Catheter Cardiovasc Intervent. (2007) 70:445–9. 10.1002/ccd.21118 17721940

[3] Carminati M Butera G Chessa M De GJ Fisher G Gewillig M. Transcatheter closure of congenital ventricular septal defects: results of the european registry. Eur Heart J. (2007) 28:2361–8. 10.1093/eurheartj/ehm314 17684082

[4] Ghosh S Sridhar A Sivaprakasam M. Complete heart block following transcatheter closure of perimembranous vsd using amplatzer duct occluder ii. Catheter Cardiovasc Intervent. (2017). 1–4. 10.1002/ccd.27177 28707408

[5] Wei Y Wang X Zhang S Hou L Wang Y Xu Y Retraction note: transcatheter closure of perimembranous ventricular septal defects (vsd) with vsd occluder: early and mid-term results. Heart Vessels (2012) 27:398–404. 10.1007/s00380-011-0153-1 21618026

[6] Butera G Carminati M Chessa M Piazza L Micheletti A Negura DG. Transcatheter closure of perimembranous ventricular septal defects: early and long-term results. J Am Coll Cardiol. (2007) 50:1189–1195. 10.1016/j.jacc.2007.03.068 17868812

[7] Yang J Yang L Wan Y Zuo J Zhang J Chen W. Transcatheter device closure of perimembranous ventricular septal defects: mid-term outcomes. Eur Heart J. (2010) 31:2238–45. 10.1093/eurheartj/ehq240 20801925 PMC2938468

[8] Yang R Kong XQ Sheng YH Zhou L Xu D Yong YH. Risk factors and outcomes of post-procedure heart blocks after transcatheter device closure of perimembranous ventricular septal defect. Jacc Cardiovasc Intervent. (2012) 5:422–7. 10.1016/j.jcin.2012.01.015 22516400

[9] Chungsomprasong P Durongpisitkul K Vijarnsorn C Soongswang J Lê TP. The results of transcatheter closure of vsd using amplatzer® device and nit occlud® le coil. Catheter Cardiovasc Intervent. (2011) 78:1032–40. 10.1002/ccd.23084 21648053

[10] Haas NA Kock L Bertram H Boekenkamp R Wolf DD Ditkivskyy I. Interventional vsd-closure with the nit-occlud®, lê vsd-coil in 110 patients: early and midterm results of the eureveco-registry. Pediatr Cardiol. (2017) 38:215–27. 10.1007/s00246-016-1502-8 27847970

[11] Qin Y Chen J Zhao X Liao D Mu R Wang S. Transcatheter closure of perimembranous ventricular septal defect using a modified double-disk occluder. Am J Cardiol. (2008) 101:1781–6. 10.1016/j.amjcard.2008.02.069 18549859

[12] Liu J Wang Z Gao L Tan HL Zheng Q Zhang ML. A large institutional study on outcomes and complications after transcatheter closure of a perimembranous-type ventricular septal defect in 890 cases. Acta Cardiol Sin. (2013) 29:271–6. 27122716 PMC4804839

[13] Lock JE Block PC Mckay RG Baim DS Keane JF. Transcatheter closure of ventricular septal defects. Circulation (1988) 78:361–8. 10.1161/01.CIR.78.2.361 3396173

[14] Bai Y Liu J Qin YW Wu H Zhao XX. Percutaneous closure of perimembranous ventricular septal defect with modified double-disk occluder: what is the outcome at 10-year follow-up? Congenital Heart Dis. (2016) 11:45–51. 10.1111/chd.12284 26171994

[15] Walsh MA Bialkowski J Szkutnik M Pawelecwojtalik M Bobkowski W Walsh KP. Atrioventricular block after transcatheter closure of perimembranous ventricular septal defects. Heart (2006) 92:1295–7. 10.1136/hrt.2005.084988 16449504 PMC1861195

[16] Butera G Gaio G Carminati M. Is steroid therapy enough to reverse complete atrioventricular block after percutaneous perimembranous ventricular septal defect closure? J Cardiovasc Med. (2009) 10:412–4. 10.2459/JCM.0b013e32832401c2 19262403

[17] Predescu D Chaturvedi RR Friedberg MK Benson LN Ozawa A Lee KJ. Complete heart block associated with device closure of perimembranous ventricular septal defects. J Thoracic Cardiovas Surgery (2008) 136:1223–8. 10.1016/j.jtcvs.2008.02.037 19026807

[18] Tucker EM Pyles LA Bass JL Moller JH. Permanent pacemaker for atrioventricular conduction block after operative repair of perimembranous ventricular septal defect. J Am Coll Cardiol. (2007) 50:1196–200. 10.1016/j.jacc.2007.06.014 17868813

[19] Saurav A Kaushik M Mahesh Alla V White MD Satpathy R Lanspa T. Comparison of percutaneous device closure versus surgical closure of peri-membranous ventricular septal defects: a systematic review and meta-analysis. Catheter Cardiovasc Intervent. (2015) 86:1048–56. 10.1002/ccd.26097 26257085

[20] Kloecker L Emmel M Sreeram N. Late complete left bundle branch block after transcatheter closure of a muscular ventricular septal defect. Cardiol Young (2010) 20:560–1. 10.1017/S1047951110000788 20836201

[21] Zuo J Xie J Yi W Yang J Zhang J Li J. Results of transcatheter closure of perimembranous ventricular septal defect. Am J Cardiol. (2010) 106:1034–7. 10.1016/j.amjcard.2010.05.040 20854970

